# Implanted injection port for routine drug delivery to the middle ear in mice

**DOI:** 10.1371/journal.pone.0349097

**Published:** 2026-05-14

**Authors:** Isabel T. Held, Niranj A. Kumar, Azmi Marouf, Kumar N. Alagramam

**Affiliations:** 1 Otolaryngology, Case Western Reserve University School of Medicine, Cleveland, Ohio, United States of America; 2 University Hospitals Ear, Nose & Throat Institute, University Hospitals Cleveland Medical Center, Cleveland, Ohio, United States of America; LSU Health Shreveport, UNITED STATES OF AMERICA

## Abstract

Small molecule drugs designed to rescue disorder-specific mutant proteins causative of progressive sensorineural hearing loss need to be delivered regularly. An example is clarin-1 N48K in Usher syndrome type IIIA (USH3A). In these cases, local delivery has significant advantages over systemic delivery. Genetic predisposition to sensorineural hearing loss may mean the invasive (surgical) approach to access the cochlea is detrimental to hearing preservation. Alternatively, delivering drugs near the round and oval window from the middle ear or tympanic cavity via a tympanostomy tube may be a viable option to treat genetic hearing loss. To test the hypothesis in a mouse model, we first must develop a way to routinely deliver the drugs to the tympanic cavity space in mice that is minimally detrimental to hearing and translatable to humans. Limitations of size and other practical considerations preclude implantation of a tympanostomy tube in mice. The goal of this pilot study was to determine feasibility and assess the impact of implanting an injection port in mice. This approach would serve as a surrogate for drug delivery through tympanostomy tubes in humans. We repurposed the Vascular Access Button (Instech Labs, PA) to access the tympanic cavity in mice that mimics hearing loss in USH3A and its wild-type counterpart. The right ear served as the non-surgical control for all mice. At 2- and 4-weeks post-operation, we monitored behavior, physical attributes, balance, and hearing. The implant was generally well-tolerated in mice. Saline injections through the port were tested for two weeks post-operation without issue, and hearing was not significantly affected. Here, we present a fully implantable method in mice for routine drug delivery to the middle ear, with minimal damage or implant-induced hearing loss. This approach could be used to investigate drug therapies in preclinical models of sensorineural hearing loss.

## Introduction

Pathogenic variants in more than 125 genes are associated with congenital or progressive hearing loss [1]. Genetic hearing loss often underlies defects in the sensory ‘hair’ cells within the cochlea [2–3]. For example, missense and/or nonsense mutations in gene coding for protein myosin 7A, protocadherin-15, and clarin-1, cause hearing loss due to defects in the hair cells of the cochlea [2,4,5]. The restoration of hearing in patients with otoferlin gene mutation using gene replacement therapy [6–7], highlights the potential of gene therapy (GT) to mitigate hearing loss associated with other genes. However, the development of alternatives to gene-based therapies to mitigate genetic causes of hearing loss, such as small molecule therapy, is important and warranted for several reasons. A few of those reasons indicated here. No treatment is available to restore or preserve hearing for most genetic hearing loss; prior exposure to adeno-associated virus (AAV) would limit the efficacy of AAV-based GT; small molecule drugs that restore (mutant) protein function and preserve hearing [8–9] could serve as the primary treatment for both ears or the untreated ear in a patient that received GT in the contralateral ear.

Small molecules that rescue disorder-specific mutant proteins causative of sensorineural hearing loss, such as clarin-1-N48K in Usher syndrome type IIIA (USH3A), need to be administered for the life of a patient at doses and intervals guided by empirical studies. Small molecules delivered closer to the cochlea are likely to be more effective with minimal toxicity compared to systemic or oral delivery. To circumvent the anatomical obstacles of the ear, including the blood-labyrinth barrier, and deliver therapeutic agents to the tympanic cavity and inner ear many approaches have been investigated in laboratory animals. This includes but is not limited to: nanoparticle systems that are injected through the tympanic membrane [10–11]; use of cochlear catheters as an implant serving as an access port to the cochlea [12]; gel-based methods, where the drug of interest is encapsulated in thermosensitive microemulsion gel or poloxamer 407-based microbubble and injected into the middle ear space [13–14]; micro-osmotic pump with an attached catheter to access the round window niche, the cochlea, or the posterior semicircular canal [15]. These approaches are potentially useful where short-term delivery is likely to have a positive impact in the long term. For example, trans-tympanic injection of otoprotective drug-infused-gel to prevent hearing loss after exposure to loud noise, or a catheter-based delivery to the cochlea to reduce inflammation and fibrosis following cochlear implantation. However, these approaches may not be viable options for chronic delivery of small-molecule drug-based therapy to preserve hearing in patients genetically predisposed to sensorineural hearing loss (SNHL), such as USH3A patients. Several critical factors need to be considered to achieve a viable, durable drug delivery approach for those with hearing loss due to mutant protein dysfunction.

First, small molecule drugs, such as BF844 and artemisinin, demonstrated to preserve hearing in animal models with hearing loss associated with USH3A causative mutation CLRN1-N48K [8,9,16], need to be administered for life. A drug encapsulated gel-based technique would require injections of the drug encapsulated gel across the tympanic membrane at regular intervals to replenish the drug. Repeated injections are likely to increase the chances of injection trauma to the tympanic membrane or infection, and patient non-compliance is more probable. Implanting a micropump-catheter device for direct access and delivery of the drug to the cochlear fluid carries potential risks. The trauma of surgery to place the catheter, post-implant response, inflammation and fibrosis, risk of infection, and need to replace the micropump-catheter prosthetic in case of malfunction exacerbate a subject’s susceptibility to SNHL and compromise the goal of preserving hearing in subjects with a genetic predisposition to SNHL. Repeated transtympanic injections to deliver therapeutic agents to treat a patient with genetic hearing loss is not a viable option long term. Alternatively, delivering drugs close to the round and oval window from the middle ear space via a tympanostomy or ventilation tube (VT) would be a viable and inexpensive option to treat genetic hearing loss without the risks and downsides of sophisticated approaches.

Using a VT in USH3A patients, for example, potentially eliminates the first barrier (tympanic membranes) for a medicated ear drop to reach the membranes covering the oval and round windows of the cochlea. Further, with a simple step – turning the patients’ head sideways for a short time while in a supine position after application of the ear drop – we can increase the exposure of the medicated drops to the surface of the round and oval window membranes and facilitate entry of the drug into the cochlea. The properties of the small molecule drug and solvent used will be important factors in the amount of medication traversing the cochlear membranes. While medicated ear drops administered through a VT means possible loss of medication via the eustachian tube and temporary discomfort, we hypothesize that the benefits of using a VT to deliver drugs proven to work in preclinical models outweigh the disadvantages.

To test the hypothesis in a genetic model of hearing loss, we first need to develop an approach for routine delivery of drugs to the middle ear space in a suitable animal model. Mice are a proven model in hearing research [17–18], like the mouse model of progressive hearing loss associated with USH3A causative mutation CLRN1-N48K [8]. Limitations of size and other practical considerations preclude implantation of a tympanostomy tube in mice. The goal of this pilot study was to determine feasibility and overall impact of implantation of an injection port in mice. We repurposed the Vascular Access Button (VAB^TM^) (Instech Labs, PA) to access the middle ear space or tympanic cavity in mice, modeling after the approach used in guinea pigs [19]. To our knowledge, this is the first time that the VAB has been used to access the middle ear space or tympanic cavity in mice. We refer to the implant as TCAP (Tympanic Cavity Access Port). We examined the tolerance of mice to TCAP implants over an extended period, including its impact on mobility, feeding, and cage behavior, and most importantly, whether the implantation of TCAP caused hearing loss in mice or exacerbated hearing loss in a mouse model of USH3A. Data from the pilot study is encouraging. The study suggests that mice implanted with TCAP could serve as surrogates for drug delivery through VT in humans and test the efficacy of drugs delivered through the middle ear space in mouse models of genetic hearing loss.

## Materials and methods

### Mice

We used two- to three-month-old C57BL/6J mice (from Jackson Labs, ME) and the mouse model for progressive hearing loss in USH3A [8]. Both males and females were used. Mice were examined for the presence of otitis media in both ears before they were used in the study.

### Implant

For the implant in this report, we repurposed the Vascular Access Button – made to access the vein for sampling or drug infusion in mice – manufactured from Instech Labs, PA. A picture of the parts is shown in the results section. Briefly, the implant materials used include the following: An injection port, with a smaller epidermal base, encased magnet, and larger subdermal wheel-shaped collar, served to strengthen and maintain the position between the shoulder blades on the dorsal side after implantation. The magnet holds the injection port cover. The curved stainless tube, from the bottom of the injection port, is attached to a catheter; the other end of the catheter is inserted into the fenestration made in the bone to access the tympanic cavity.

### Hearing assessment

Baseline hearing was assessed by auditory-evoked brainstem response (ABR) recordings using Intelligent Hearing Systems software and hardware (Miami, FL). The presence of an ear infection (otitis media) will confound the assessment of the impact of the implant on hearing. Prior to every ABR recording, the tympanic membrane of the ears was examined to rule out otitis media. The protocol and equipment used for ABRs are detailed elsewhere [20]. Briefly, mice were anesthetized by an intraperitoneal injection of ketamine hydrochloride and xylazine hydrochloride as described previously [20]. To ensure middle ear integrity, anesthetized mice were screened for earwax, otitis media, and tympanic membrane rupture using a wall-mounted Zeiss microscope (used for rodent surgical procedures) immediately before every ABR recording. After ruling out middle ear issues, the mouse was transferred to a designated wooden box where ABR recordings were carried out. Mice were placed on a thermal pad to maintain body temperature during the procedure. Subdermal needle electrodes were placed at the scalp vertex, the mastoid region under the left ear, and the mastoid region under the right ear. Sound stimuli were presented via tubing inserted into the ear, and ABRs were recorded in closed-field conditions in a chamber to minimize interference from ambient noise. Parameters were set at 1,024 sweeps per run, starting at stimulus intensity 90 dB SPL, followed by a reduction in 10 dB SPL steps until the hearing threshold was reached. We administered pure tones at 8, 16, and 32 kHz that represent the low, medium, and high frequencies, respectively, along the tonotopic gradient of the mouse cochlea. The lowest stimulus level that yielded a detectable ABR waveform at 8, 16, and 32 kHz was defined as the threshold.

### Pre-training before implantation

Mice were housed in microisolator cages, which included a metallic (non-magnetic) lid that holds food pellets and a water bottle. The lid is forged with a depression to hold food and a water bottle, and the downward slope of the depression increases access to food and water inside the cage. However, during recovery from surgery (3–4 days following implantation), it would be important to provide ready access to food and hydration and eliminate obstructions in the cage that could dislodge the injection port unit (to be placed on the dorsal side later) affecting healing and integration of the unit as the animal moves around the cage. After recording baseline ABRs and before surgery/implantation of the injection port, mice were transferred to cages with flat top plastic lids, Clear H_2_O Hydrogel (from ClearH_2_O, Inc., ME), for hydration and food pellets. We found that pre-training mice in the modified cage setup before surgery (implantation) increased the chance of mice using the hydrogel post-surgery and enhanced survival. A few days after surgery, mice were returned to standard cages with hydrogels included as a secondary source of hydration.

### Surgery

The mice were anesthetized with a ketamine/xylazine mixture via IP injection and placed on a heating blanket. A post-auricular incision was made by the left ear, and the bulla was exposed. A 2 mm hole was made into the tympanic cavity bone with a 26-gauge needle. A 26-gauge catheter (provided in the kit from Instech Labs, PA) was cut to reach from the tympanic cavity to the shoulder blades, then inserted into the hole and secured in place with tissue and veterinary glue. An additional incision was made between the shoulder blades, and the top skin was separated from the tissue to create a “pocket.” Specifically, a small pocket of the epidermis was teased apart from the dermis below and the disc (base flange) of the injection port was inserted. The other end of the catheter was passed underneath the skin to reach the second incision between the shoulder blades. Here, the catheter was connected to the stainless-steel tube at the bottom of the injection port button. The subdermal wheel-shaped flange of the injection port unit was inserted into the skin pocket, with the injection port and epidermal base placed above the skin. That location on the dorsal side of the mouse was selected for implanting the injection port because the mouse cannot reach that part of its body with its mouth or hind legs and dislodge the port. After insertion of the subdermal flange, incision sites were then sutured, with only the injection port visible. Ten µL of saline solution was injected through the port to prevent potential clogging of the catheter. After that, the cap (included in the kit) was placed on the injection port to align with the magnet at the base of the port to close it and cover the magnet. When the injection port is not capped, during implantation surgery or during an injection post-implantation, it is important to either keep metal surgical devices, including forceps, away from the port’s magnet or to use surgical instruments made of non-magnetic material. Attempts to detach a forceps that accidentally attached to the magnet could potentially dislodge the entire injection port after implantation, so we would advise handling this with care. After completion of the implantation surgery, mice received an injection of Lidocaine to serve as a pain medication immediately after surgery. After the injection, the mice were left to recover on a heating pad and eventually returned to their cages with cage mates with or without an implant. Post-surgery survival and recovery are better when the mice are returned to the cage. Steps in the surgical procedure are illustrated in the results section.

### Post-operation handling

To restrain a mouse for injections or other purposes, it is necessary to pick them up by their tail and scruff them. Because the implant is close to the neck on the dorsal side, restraining implanted mice has the potential to dislodge the implant. To avoid dislodging the implant during post-operational handling and injections, mice were mildly sedated for a short duration (~2–3 minutes) using the isoflurane bell jar method [21]. On each of those 3 days post-surgery, mice were observed for any physical signs of pain or inflammation. Additionally, they were treated with an injection of Meloxicam for any potential pain, as well as an injection of Enrofloxacin, an anti-bacterial medication. If necessary, we also applied an anti-bacterial cream to the suture sites at any sign of inflammation. After 3 days of post-operational care, mice were still observed daily for any signs of distress. The hydrogel stayed in the cage as a secondary option for hydration. An additional step we took to enhance survival post-surgery was to house implanted mice with unoperated cage mates. We did this for both males and females, as we noted that physical attention from the littermates, as opposed to isolated housing, seemed to aid in the recovery process. Remarkably, unoperated littermates socialized normally and did not bite or dislodge the injection ports in the operated littermates.

### Statistical approach

To evaluate the effects of TCAP implantation on ABR thresholds over time, we constructed linear mixed effects models incorporating ear (left, right), time post-procedure (baseline, 2 weeks, 4 weeks), and their interaction as fixed effects. A random intercept for mouse was included to account for the paired and repeated-measures structure of the data, as ABR thresholds were recorded from both ears of each animal at multiple time points. This modeling approach enabled assessment of whether changes in hearing thresholds over time were attributable to the implant, while accounting for within-animal correlation and baseline variability. Separate models were fit for each tested frequency (8 kHz, 16 kHz, 32 kHz).

### Ethical statement of animal use

All animal procedures used in this study were approved by the Case Western Reserve University (CWRU) Institutional Animal Care and Use Committee (IACUC) (Approved Protocol number 2016−0258) in writing. All procedures and animal handling complied with NIH ethics guidelines and approved protocol requirements of the IACUC at CWRU. Animals were housed in the Health Science Animal Facility (HSAF) at Case Western Reserve University, a climate-controlled facility where animals are housed in microisolator cages with access to sterilized food and water. All the experimental procedures were designed to minimize pain and distress, such as the use of anesthesia where appropriate.

## Results

**Fig 1:**
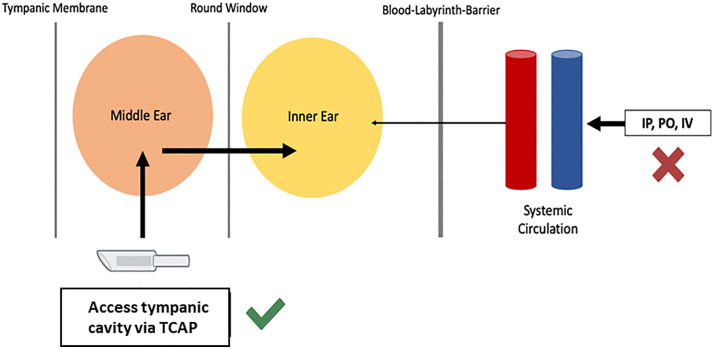
A schematic the rationale and approach. This approach would allow for delivery to the tympanic cavity with diffusion through the round window to the inner ear, bypassing the blood-labyrinth barrier (BLB)..

**Fig 2:**
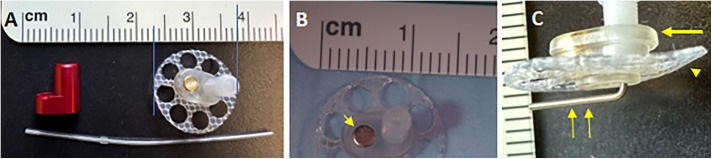
Parts of the implant as provided by Instech Laboratories. **A**. The entire set: injection port unit, magnetic cap (red), and catheter. **B**. Close-up of the injection port and the embedded magnet (arrowhead). **C**. Side-view of the injection port unit showing smaller epidermal base (arrow), a larger subdermal wheel-shaped flange (arrowhead), and a metal tubing (double arrow) to connect the injection port to the catheter.

**Fig 3:**
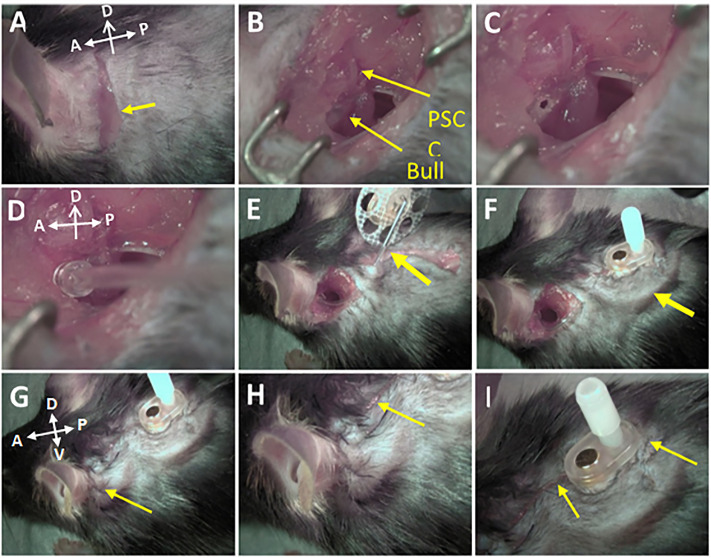
Visualization of the surgery process. **A.** the initial incisions (arrow), **B.** exposing the PSCC and bulla, **C.** fenestration in the bulla, **D.** attaching the catheter to the bulla, **E.** attaching the injection port, **F.** suturing the area around the button, **G.** suturing the remaining incision, **H.** and **I.** close-up on completion of surgery. Anatomical orientation noted in panels A, D, and G represents the anatomical orientation of the other panels in the same row.

Fig 1 illustrates the rationale and approach for routine drug delivery to the middle ear (tympanic cavity) in mice—a well-established mammalian model in hearing research. The Tympanic Cavity Access Port (TCAP) comprises an injection port unit, a magnetic cap to seal the orifice and shield the internal magnet, and a catheter (Fig 2). The multi-step surgical implantation process (Fig 3) was optimized over the course of a year to ensure a consistent outcome and procedure time of 45–60 minutes.

### No significant signs of distress associated with TCAP implantation

**Fig 4:**
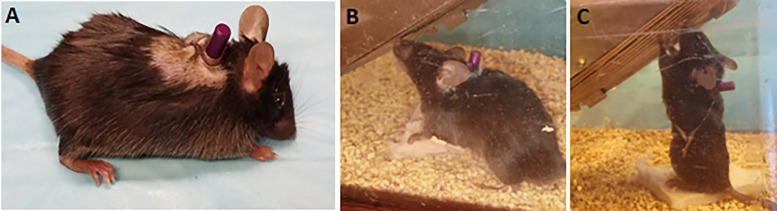
The position of TCAP and post-implantation posture and mobility. **Panels A – C** illustrate the following points **A.** A picture taken immediately after the implantation surgery shows the injection port mounted dorsally between the shoulder blades**. B** and **C.** Still frames from associated video (supplemental material) recorded several days after implantation, showing a mouse displaying normal posture and the ability to reach for food standing on two legs.

One day after TCAP implantation, both wildtype and USH3A mice showed no signs of head bobbing or tilting, or circling behavior, indicating that the vestibular sensory system was not damaged in the implantation procedure. Additionally, we observed no otitis media, excessive scratching of the surgical sites, wound infections, or swelling of the tissue adjacent to the incision sites. This was all confirmed during daily post-operational checks. We found no increase in earwax accumulation in or around the ear canal, and the implanted mice displayed normal grooming and feeding behavior comparable to the un-implanted siblings. Overall, the implanted mice remained agile with little to no difference in mobility compared to their unoperated cage mates (Fig 4;). The implanted mice co-existed with their littermates as they did pre-implantation, and the un-implanted sibs show no indication that they were averse to the implanted sibs in the cage. These observations indicated that TCAP implantation did not have a negative impact on survival or normal activity and mobility of these mice. Lastly, following one week and two weeks post-implantation, 10–20 µL of saline was injected through the catheter with no issues, indicating no clogging of the catheter up until that point. Prior to injection in mice implanted with the TCAP, we estimated an initial syringe volume of 10–20 µl. By injecting saline into a catheter attached to a cadaver mouse and measuring the volume retained post-injection, we estimated the maximum tympanic cavity volume in the adult C57BL/6J strain used in this study to be between 6 and 8 µl. In mice implanted with the TCAP device, we injected 10–20 µl of saline. We estimate that 6–8 µl immediately filled the tympanic cavity, while the remainder was retained within the catheter and device. Because subsequent injections were unimpeded and no leakage was observed at the port, we assume the retained volume transitioned into the tympanic cavity passively over time. Post-operative microscopic inspection and ABR recordings confirmed that these injections caused no damage to the tympanic membrane or ossicular chain, as hearing thresholds remained stable.

### No significant hearing loss associated with TCAP implantation

The implanted mice co-existed with their littermates as they did pre-implantation, and the unimplanted sibs show no indication that they were averse to the implanted sibs in the cage. Further, throughout the study, we noticed a distinct startle response was observed in both implanted and control mice whenever the cages were closed. This sound, produced by placing the metallic lid on the polycarbonate base, suggests that TCAP implantation did not cause deafness in wildtype mice or exacerbated hearing loss in USH3A mice. However, as the startle response is not bilaterally dependent, a definitive assessment of unilateral hearing loss would require separate quantitative analyses for each ear.

For a quantitative assessment of hearing in mice across the study period, we recorded and analyzed auditory-evoked brain stem response (ABRs). Before ABR recording was initiated, each animal was observed for otitis media in both ears. This served two purposes: one, to determine whether the fenestration of the tympanic cavity bone and the insertion of a catheter resulted in mice susceptible to infection and otitis media compared to the unoperated contralateral ear or unoperated mice in the same cage. Second, in rare instances mice could acquire otitis media for reasons independent of the surgery but, if present, will confound our investigation of whether implantation induces hearing loss. We examined every mouse at various stages in the study and did not observe any sign of otitis media in either the left or right ear in any of the implanted wild type or USH3A mice or the unoperated siblings. Therefore, any significant ABR threshold elevation associated with the implanted ear compared to the baseline would reflect an implant-induced hearing loss*.*

**Fig 5:**
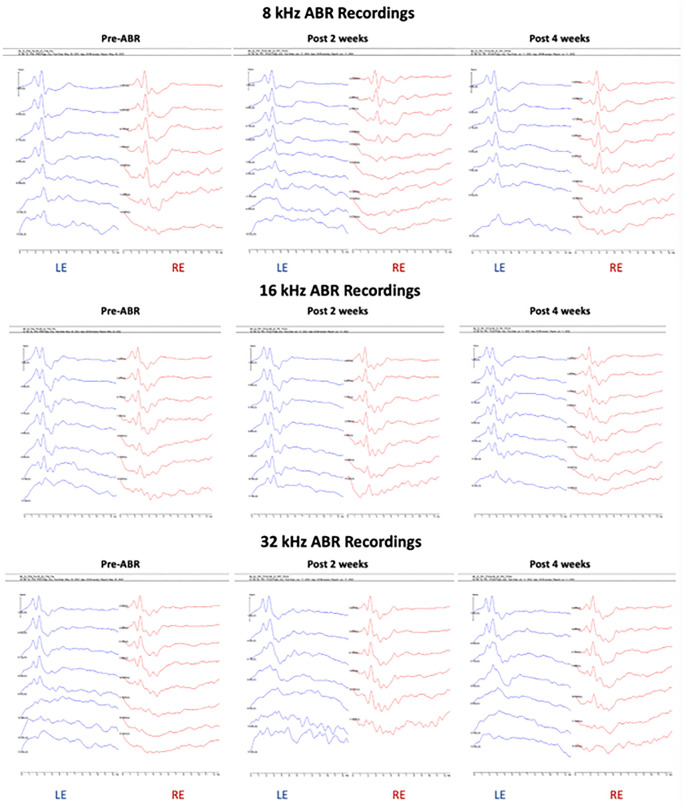
ABR recordings from mouse # 2 (Table 1). Waveforms from pre-surgery, post-surgery 2 weeks, and post-surgery 4 weeks, shown here. TCIP implanted in the left (LE); right ear (RE) served as the control ear. It should be noted that at 8 kHz, the ABR waveform in the un-implanted right ear (RE) 2 weeks post implantation (post 2 weeks) suggest possible hearing loss, but it is likely an artifact of displaced tube placed in the ear to deliver sound stimuli, because the RE from the same mouse at 4 weeks post implantation (post 4 weeks) showed ABR waveforms and thresholds comparable to pre-ABR (pre-implantation).

**Fig 6:**
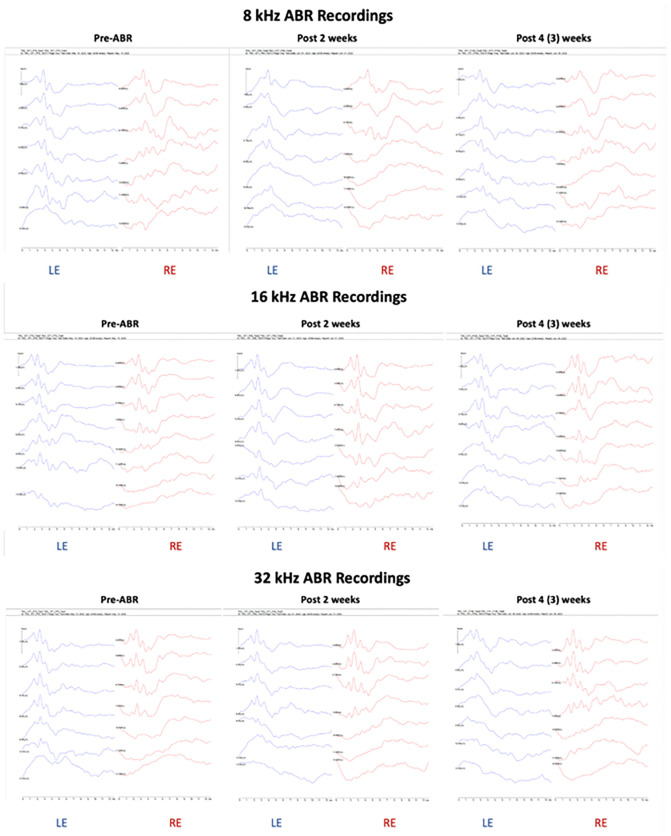
ABR recordings from an USH3A mouse #6 (Table 1). Waveforms from pre-surgery, post-surgery 2 weeks, and post-surgery 4 weeks, shown here. Surgery performed in the left ear (LE); the right ear (RE) served as the control ear.

After the initial baseline ABR recording pre-surgery (under Materials and Methods), we repeated this procedure at 2- and 4-weeks post TCAP implantation. Table 1 displays the raw ABR threshold data, illustrating the range of outcomes following TCAP implantation. Representative ABR waveforms for wild-type and USH3A mice (Figs 5 and 6, respectively) demonstrate instances of minimal hearing loss. In the wildtype mouse, the ABR waveforms at 8 and 16 kHz in the implanted left ear (LE) at 2- and 4-weeks post-implantation were comparable to the un-implanted right ear (RE) at 2- and 4-weeks, respectively, and to the pre-implantation ABR (Fig 5). However, ABR waveforms at 32 kHz showed less discrete peaks, I-III at 40-, 50- and 60-dB SPL at 2- and 4-weeks post implantation, reflecting lower peak amplitudes and a trend toward ABR threshold shifts (Fig 5). In the USH3A mouse, ABR waveforms at 8 and 16 kHz from the implanted and control ears at 2- and 4-weeks post-implantation were comparable (Fig 6). ABR waveforms at 32 kHz were comparable between the ears at 2 weeks post implantation but showed less discrete peaks in the implanted ear at 40-, 50- and 60-dB SPL at 4-weeks post implantation, reflecting lower peak amplitudes and a trend toward ABR threshold shifts (Fig 6). In contrast to the wildtype mice, USH3A mice are genetically predisposed to developing hearing loss, as reflected by the ABR waveform patterns in the un-implanted right ear at frequencies tested, suggesting that the TCAP-implant-induced hearing loss above background was negligible at 8 and 16 kHz and modest at 32 kHz. This inference is supported by raw and average ABR thresholds from the implanted left ear and un-implanted right rear recorded across the study period (Tables 1 and 2).

**Table 1:** Raw ABR thresholds from an individual mouse at 8, 16, and 32 kHz. Hearing test time points were pre-implantation, 2- and 4-week post-implantation, designated ‘Pre’, ‘Post 2’ and ‘Post 4’, respectively. The TCAP left ear (LE) was implanted; the right ear (RE) served as the control ear. The table shows data from four wild-type (numbers 1-4) and two USH3A (numbers 4 & 5) mice. Column 2 indicates age of individual mouse days postnatal (P) at various time points during the experiment.

Mouse	Postnatal Age	Time Point	LE 8 kHz	RE 8kHz	LE 16 kHz	RE 16 kHz	LE 32 kHz	RE 32 kHz
**1**	**P39**	**Pre**	30	30	30	30	30	30
**2**	**P39**	**Pre**	30	30	30	30	35	35
**3**	**P52**	**Pre**	30	30	30	30	35	40
**4**	**P52**	**Pre**	30	30	30	30	30	30
**5**	**P95**	**Pre**	40	40	30	30	50	40
**6**	**P79**	**Pre**	40	40	30	30	40	40
**1**	**P58**	**Post 2 Wks**	45	30	30	30	50	40
**2**	**P67**	**Post 2**	35	35	30	30	40	40
**3**	**P77**	**Post 2**	30	30	35	35	50	65
**4**	**P77**	**Post 2**	60	30	40	40	60	40
**5**	**P108**	**Post 2**	40	40	30	30	50	50
**6**	**P99**	**Post 2**	40	40	30	30	40	40
**1**	**P78**	**Post 4**	45	35	30	35	60	60
**2**	**P91**	**Post 4**	30	30	30	30	40	40
**3**	**P91**	**Post 4**	60	60	70	70	65	80
**4**	**P91**	**Post 4**	60	50	60	50	60	70
**5**	**P115**	**Post 4**	40	50	50	50	80	80
**6**	**P106**	**Post 4**	40	40	30	30	40	40

**Table 2:** Combined analysis of ABR thresholds of mice in this study across the study period. Mean ABR threshold across the study period for the left ear (TCAP implanted) and right ear (un-implanted control) (column 2); pre-implantation (Pre) time point (column 3); two weeks post- implantation (Post 2) (column 4); four weeks post-implantation (Post 4) (column 5).

Characteristic	Overall, N = 6	Baseline, N = 6	Post 2 weeks, N = 6	Post 4 weeks, N = 6
**8kHz**	**dB SPL ± SEM**	**dB SPL ± SEM**	**dB SPL ± SEM**	**dB SPL ± SEM**
Left Ear	40.3 ± 10.5	33.3 ± 5.2	41.7 ± 10.3	45.8 ± 12.0
Right Ear	37.2 ± 8.8	33.3 ± 5.2	34.2 ± 4.9	44.2 ± 11.1
**16kHz**				
Left Ear	35.8 ± 11.9	30.0 ± 0.0	32.5 ± 4.2	45.0 ± 17.6
Right Ear	35.6 ± 10.8	30.0 ± 0.0	32.5 ± 4.2	44.2 ± 15.6
**32kHz**				
Left Ear	47.5 ± 13.4	36.7 ± 7.5	48.3 ± 7.5	57.5 ± 15.4
Right Ear	47.8 ± 16.0	35.8 ± 4.9	45.8 ± 10.2	61.7 ± 18.3

To assess the relationship between measured hearing thresholds, duration post procedure (week: pre-implantation, 2 weeks post implantation, 4 weeks post implantation) and ear (side: left, right), we constructed linear mixed effects models evaluating the main and interactive effects of time and ear, with the random effect of mouse irrespective of its genotype (accounting for repeated/longitudinal measures within an individual mouse). We replicated this framework for thresholds recorded at each of three frequencies (8 kHz, 16 kHz, 32kHz; separate model for each frequency). No significant interaction between time and ear was observed at 8, 16 or 32 kHz (Table 3). We detected a significant main effect of time, but no effect of ear (Table 3). These results suggest that, across frequencies tested, the observed ABR threshold shifts over the study period occurred regardless of tested ear (implanted or un-implanted). These results show that the observed ABR threshold elevation was associated with factors (discussed below) unrelated to the TCAP implantation.

**Table 3:** Statistical approach. Linear mixed effects model evaluating the relationship between hearing threshold, and the main and interactive effects of time (Week) and ear tested (L, left; R, right), and the random effect of mouse.

Characteristic	Beta	95% Cl	p-value
**8kHz**			
**Time**	3.1	0.70, 5.6	0.014
**Ear**			
**L**	---	---	
**R**	−2.2	−11, 6.6	0.6
**Time x Ear**	−0.42	−3.9, 3.0	0.8
**16kHz**			
**Time**	3.8	1.3, 6.2	0.004
**Ear**			
**L**	---	---	
**R**	0.14	−8.8, 9.1	>0.9
**Time x Ear**	−0.21	−3.7, 3.3	>0.9
**32kHz**			
**Time**	5.2	2.6, 7.8	<0.001
**Ear**			
**L**	---	---	
**R**	−2.2	−12, 7.4	0.6
**Time x Ear**	1.2	−2.5, 5.0	0.5

**Fig 7:**
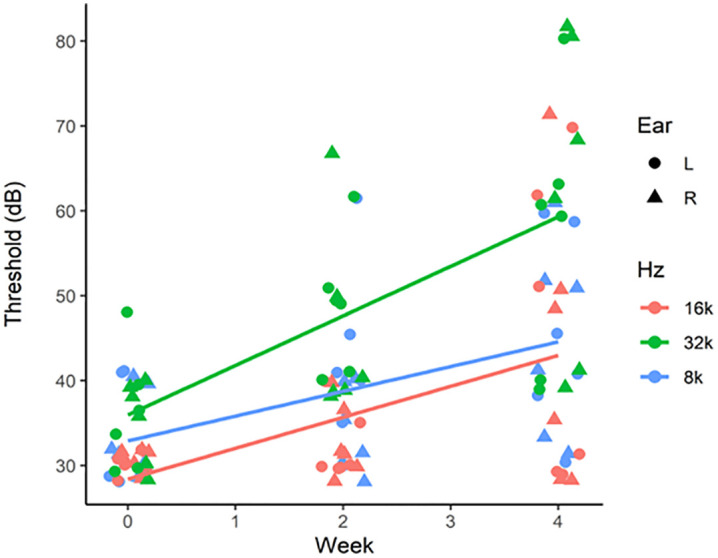
Graphical representation of trends between hearing threshold and time at various pure tone frequencies tested. Trends between hearing threshold (y-axis) and study week (x-axis) at 8 kHz (blue), 16 kHz (red), and 32 kHz (green). Implanted left ear (circle); un-implanted right ear (triangle). N = 6 (4 wildtype, 2 USH3A).

In this study we evaluated the feasibility of repurposing the available Vascular Access Button^TM^ (Instech Labs, PA) designed for mature adult mice as a conduit to access the middle ear space in mice. To fit the size of the available device, the injection port in particular, we chose to use mice that were P40-P60 for the surgery. Given this age, the mice were P70-P90 at the 4-week post-surgery recording timepoint. Both wildtype and USH3A mice used in this study are in a genetic background (C57BL/6J) susceptible to progressive loss of hearing with age, as reflected by the distribution of the mean ABR thresholds in the implanted left ear and un-implanted right ear (Table 2, and Fig 7).

One initial concern was that the magnet in the injection port (Fig 2B) might interfere with the ABR recording, as the electrodes used to record ABRs are metal and magnetic, and based on the proximity of the electrodes placed subdermal on the scalp and near the ears. However, based on the comparison of pre- and post-implantation recordings in the un-implanted ear in the wild-type mice at 16 kHz (Fig 5), for example, there is no indication that the magnet in question affects ABR recordings.

### Tissue integration of catheter and implant as shown by postmortem dissection

**Fig 8:**
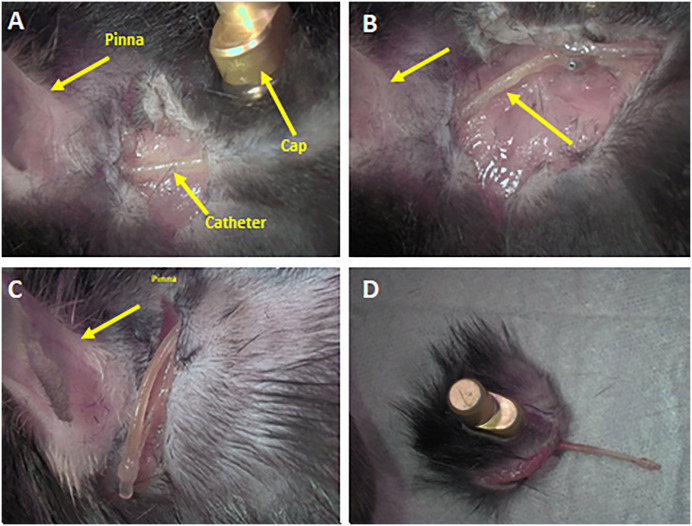
Post-mortem dissection of TCAP implanted mice at 6 weeks post implantation. **A.** Post-auricular incision showing catheter integrated into surrounding tissue, **B.** Expanded view of post-auricular incision with integrated catheter, **C.** Catheter removed from bulla hole showing tissue having grown around it, **D.** Removed injection port showing tissue grown through and around it, having fully integrated it.

Postmortem examination of mice 10–12 weeks after TCAP implantation (6–8 weeks after the last ABR recording at 4-weeks post-TCAP-surgery), showed no obvious adverse effects of subdermal insertion of the catheter. The catheter appeared to be well integrated with the surrounding tissue. In fact, a closer examination showed that the catheter was ensheathed by tissue and firmly integrated (Fig 8A). The ensheathed tissue had to be cleared to expose the catheter (Fig 8B). Postmortem observation also showed that the catheter stayed attached to the insertion site in the bone covering the tympanic cavity, and the base of the injection port between the shoulder blades at the other end*.* The skin around the injection port on the dorsum healed and sealed the base of the injection port without any obvious signs of detachment or incompatibility (Fig 8D). We do note that some clogging of the catheter end by a yellow substance was noticed during this post-mortem dissection. Possibly, some interstitial fluid or fluid from the middle ear cavity may have entered the catheter and clogged it (clogging occurred in 4 out of the 6 mice). However this did not occur until about 4 weeks after the surgery. Mice were maintained for 6–8 weeks after the 4-week post-surgery ABR recording for long-term observation/effects in mice, as TCAP-implanted mice could serve as a model to test the efficacy of chronic drug treatment. We can note that the injection port remained in place for several months, until the mice were sacrificed for post-mortem analysis, and the implant did not affect the movement or survival ability of the mice in any way. While ABR data was collected over 4 weeks, the mice were observed for 10–12 weeks post-implantation before postmortem analysis. We acknowledge that this timeframe remains relatively short for a chronic device; however, as an exploratory feasibility study, we believe this duration is sufficient to demonstrate initial tolerability and tissue integration. This work establishes the foundation for future long-term studies, which will utilize significantly longer observation periods.

## Discussion

Our goal was to develop an implant in mice that could serve as a surrogate for the delivery of otoprotective drugs through tympanostomy tubes in humans. Once developed and proven to be well tolerated in mice, the implant can be used in future studies to test the efficacy of promising small-molecule candidates. It would be administered through the middle ear space to preserve hearing in genetic models of hearing loss. We repurposed the Vascular Access Button (VAB^TM^) (Instech Labs, PA) to access the middle ear space or tympanic cavity in mice, modeling after the approach used in guinea pigs [19]. To our knowledge, this is the first time the VAB has been used to access the tympanic cavity in mice.

We present a fully implantable method in mice for routine delivery of fluids to the tympanic cavity or middle ear space, with minimal damage or implant-induced hearing loss in wild-type mice and in a mouse model of genetic hearing loss. We refer to the implant as TCAP (Tympanic Cavity Access Port). Over an extended period, we examined the tolerance of mice to TCAP implants, including its impact on mobility, feeding, cage behavior, and, most importantly, whether the implantation of TCAP caused hearing loss in mice or exacerbated hearing loss in a mouse model of USH3A. The results suggest that mice implanted with TCAP could serve as surrogates for drug delivery through tympanostomy tubes in humans. This would allow us to test the efficacy of drugs delivered through the middle ear space in mouse models of genetic hearing loss.

To determine injection parameters for mice implanted with TCAP, we estimated a syringe volume of 10–20 µl. The adult mouse tympanic cavity volume was estimated at 6–8 µl, consistent with previous literature [22–23]. While this small volume raises concerns regarding the delivery of a sufficient therapeutic dose for cochlear hearing loss, several factors suggest that TCAP (in mice) and PE tubes (in humans) can facilitate effective otoprotection. First, local delivery requires significantly lower drug concentration than systemic administration. Second, the adult mouse cochlear fluid volume of ~2.5 ± 0.1 µl [24–25] is three to four times smaller than the tympanic cavity, allowing for a favorable concentration gradient. Third, the implanted injection port enables repeated dosing to overcome volume constraints. Fourth, selection of agents that are actively transported across the tympanic membrane [26]. These principles translate well to humans, where the cochlear volume (83–100 µl) is vastly outweighed by the tympanic cavity volume (500–1000 µl) [27–28], likely accommodating drugs with diverse pharmacokinetic profiles.

There are much more complex and technically advanced devices reported for tympanic cavity drug delivery, as described in Magdy et al. 2022 [29]. In contrast, our approach shows an alternative for drug delivery that does not require a lot of resources. We show that TCAP is well tolerated overall, with minimal post-implant effects on hearing and overall well-being of the mice, including the transgenic USH3A mouse model used here that are genetically predisposed to hearing loss. Though not significant, we did notice some hearing loss associated with TCAP implantation. It should be noted that interpretation of the results in this investigation is based on mice in the C57BL/6J genetic background, which are susceptible to age-related hearing loss [30–32]. Response to the implant procedure may vary in other genetic backgrounds. Mouse strains including the CBA/CaJ and CAST/Ei with no known susceptibility to hearing loss may prove to be better strains for TCAP implantation. Future studies will be necessary to determine the impact of TCAP implantation in mice from different genetic backgrounds.

From a translational viewpoint, this approach (implanting a catheter) might turn out to be quite invasive if performed in human patients; however, we present this approach with that in mind, providing an opportunity to test drug delivery in animal models, rather than perfecting a human delivery approach. Ideally, a drug or medication for long-term treatment of chronic conditions can be developed using our approach, and then it can be delivered through tympanostomy tubes as a humane, less-invasive equivalent. With the TCAP implant, we were able to inject saline (10–20 µL) for up to 2 weeks post-surgery without any issue. Around 3–4 weeks post-operation, more pressure had to be applied while injecting, suggesting possible blockage somewhere in the catheter. Post-mortem examination 10–12 weeks after TCAP implantation (6–8 weeks post-final ABR recording) revealed that four of the six catheters were obstructed by a yellow substance. Notably, routine saline flushing had been discontinued several weeks prior to this analysis. To mitigate future clogging, researchers could implement frequent flushing with heparinized saline throughout the animal’s lifespan or utilize catheters with specialized polymer coatings designed to resist bacterial adhesion, biofilm formation, and encrustation [33].

Further investigation will focus on optimizing the surgical approach, as well as significantly increasing our sample sizes, both control and experimental mice. Additionally, we would like to test TCAP implant in other mouse strains not predisposed to age-related hearing loss to see if the strain background has an influence on the outcome. Our short-term goal is to focus on further optimizing the saline injections, while our long-term goal encompasses eventually moving on to injecting Gentamicin to show further proof of concept. Ideally, we would like to eventually inject treatments for USH3A into mice.

Our study does have a few limitations, one of them being that the mice used, have a genetic predisposition to hearing loss due to their background. Using a mouse with a different background could lead to more normal data distribution and lower hearing threshold, though it should not change the conclusion of the study that TCAP implantation is well tolerated and it does not induce significant hearing loss. Another potential limitation is that the mice need to be a certain size and age (at least 2 months old) before the implant can be surgically placed on the mouse; drug administration prior to the implantable age must be via an alternate route (IP or oral gavage) in mice. This limitation will not have an impact from a translational perspective, because a PE tube or tympanostomy tube can be placed in babies as young as 6 months old. Even though further testing and optimization is necessary, we believe that the TCAP approach could be used to investigate drug therapies in preclinical models of sensorineural hereditary hearing loss and provide a feasible and relatively easy approach to the drug delivery method.
